# Investigating the effect of carbon source on rabies virus glycoprotein production in *Pichia pastoris* by a transcriptomic approach

**DOI:** 10.1002/mbo3.489

**Published:** 2017-05-18

**Authors:** Safa Ben Azoun, Héla Kallel

**Affiliations:** ^1^ Laboratory of Molecular Microbiology Vaccinology and Biotechnology Development Biofermentation Unit Institut Pasteur de Tunis Université Tunis El Manar Tunis Tunisia

**Keywords:** carbon source, cell physiology, glucose, methanol, *Pichia pastoris*, rabies virus glycoprotein

## Abstract

Several factors affect protein expression in *Pichia pastoris*, one among them is the carbon source. In this work, we studied the effect of this factor on the expression level of rabies virus glycoprotein (RABV‐G) in two recombinant clones harboring seven copies of the gene of interest. The expression was driven either by the constitutive glyceraldehyde‐3‐phosphate dehydrogenase (GAP) promoter or the inducible alcohol oxidase1 *(* AOX1) promoter. Clones were compared in terms of cell physiology and carbon source metabolism. The transcription levels of 16 key genes involved in the central metabolic pathway, the methanol catabolism, and the oxidative stress were investigated in both clones. Cell size, as a parameter reflecting cell physiological changes, was also monitored. Our results showed that when glucose was used as the sole carbon source, large cells were obtained. Transcript levels of the genes of the central metabolic pathway were also upregulated, whereas antioxidative gene transcript levels were low. By contrast, the use of methanol as a carbon source generated small cells and a shift in carbon metabolism toward the dissimilatory pathway by the upregulation of formaldehyde dehydrogenase gene and the downregulation of those of the central metabolic. These observations are in favor of the use of glucose to enhance the expression of RABV‐G in *P. pastoris*.

## 1 Introduction

*Pichia pastoris* (syn. *Komagataella* sp.) has become one of the most popular platforms for recombinant protein expression; this system outperforms other competitive yeasts such as *Saccharomyces cerevisiae,* for foreign protein production (Puxbaum, Mattanovich, & Gasser, 2015). The importance of *P. pastoris* stems from the high cell density level that can be achieved during bioreactor culture and the low level of host cell proteins secreted into the culture medium, among others (Ahmad, Hirz, Pichler, & Schwab, 2014). However, low production levels are still observed for some proteins. In general, the metabolic burden induced upon heterologous protein production in yeasts has a detrimental effect on cell growth, as reported by Nocon et al. (2014) who explained that by the attribution of the generated energy to product synthesis instead of biomass accumulation.

Heterologous protein expression in yeasts can be affected by different factors. In *P. pastoris* potential limiting factors of foreign protein expression are gene dosage (Shen, Ming, Hai‐Bin, Hua, & Shu‐Qing, 2012), efficient transcription of the transgene using strong promoter (Gasser et al., 2013), protein folding in the reticulum endoplasmic (RE) (Vanz, Nimtz, & Rinas, 2014), and protein secretion (Pfeffer et al., 2011). Additionally, bioprocess parameters such as pH, temperature, growth rate, and substrate type also affect protein expression in *P. pastoris* (Dragosits et al., 2009; Files, Ogawa, Scamanb, & Baldwina, 2001; Xie, Zhou, Du, Gan, & Ye, 2004). However, only few studies were focused on the influence of heterologous protein expression on yeast metabolism (Baumann et al., 2010; Çelik, Çalik, & Oliver, 2009; Nocon et al., 2014; Prielhofer et al., 2015; Xie et al., 2004).

Prielhofer et al. (2015) studied the transcriptional and translational profiles of *P. pastoris* cultivated in shake flasks under four bioprocess conditions: (1) excess of glycerol, (2) excess of glucose, (3) limiting glucose concentration, and (4) methanol induction conditions. They showed that the carbon source affects differently, the transcription level of various endogenous genes; however, cells grown on an excess of the carbon source (glucose or glycerol) showed comparable transcriptome. They also found that the synthesis of ribosome components was not affected by methanol despite the low growth rate depicted by the cells grown under this condition. Other studies (Inan & Meagher, 2001; Xie et al., 2004) showed that the carbon source also affects the expression of heterologous genes besides endogenous genes. Xie et al. (2004) reported that different carbon sources like acetate, glycerol, sorbitol, and lactic acid used during the cultivation of recombinant *P. pastoris* displayed different effects on angiostatin production level. The highest angiostatin production level was achieved when lactic acid or sorbitol were used. Other carbon sources such as mannitol, alanine, and sorbitol have also been tested for the production of β‐galactosidase by recombinant *P. pastoris* Mut^‐^ clones (Inan & Meagher, 2001). All these carbon sources were able to improve β‐galactosidase production level as compared to glucose or glycerol, and to reduce the amount of methanol required for the expression of the heterologous protein. The use of mixed substrates can have some attractiveness when setting up the process at large scale; it can reduce the quantity of methanol, and therefore the risk associated with the storage of large amounts of this product, and consequently can contribute to reduce the overall cost.

The carbon source can also affect the intracellular amount of the heterologous protein, even if the expression is even if the protein is secreted. In this line, Hohenblum, Gasser, Maurer, Borth, and Mattanovich (2004) demonstrated that recombinant trypsinogen level retained in *P. pastoris* cells was dependent on the carbon source but not on the promoter.

In previous studies, we generated two recombinant clones of *P. pastoris* KM71H Mut^S^ harboring seven copies of the rabies virus glycoprotein (RABV‐G) gene (Ben Azoun, Belhaj, Göngrich, Gasser, & Kallel, 2016; Ben Azoun, Belhaj, & Kallel, 2016). The expression of the target protein was driven either by AOX1 promoter (aox7) or GAP promoter (gap7) and directed in both clones to secretion by the alpha mating factor of *Saccharomyces cerevisiae*. Expression levels of RABV‐G obtained by these clones were compared and a significant difference was seen in extracellular and intracellular compartments. We showed that this difference was obviously substrate and not promoter‐dependent as mRNA levels of RABV‐G gene were comparable (Ben Azoun, Belhaj, Göngrich, et al., 2016; Ben Azoun, Belhaj, & Kallel, 2016).

In this work, we aim to obtain deeper insights about the expression of RABV‐G in *P. pastoris*; we analyzed cell morphology and the transcriptional responses of cells grown in methanol and glucose‐containing media. A comparative transcriptional analysis was performed with several key genes used as indicators of the physiological state of recombinant *P. pastoris* clones to determine the effect of carbon metabolism on the production of RABV‐G in this yeast.

## 2 Experimental Procedures

### 2.1 Strains and media

*P. pastoris* KM71H (Invitrogen, CA, USA) was used in this study. Optimized RABV‐G gene (Genbank accession number **KT878717**) was used for the construction of the expression cassette. The generation of multi‐copy clones used in this work (gap7, aox7) was previously described in details (Ben Azoun, Belhaj, Göngrich, et al., 2016; Ben Azoun, Belhaj, & Kallel, 2016); these clones were constructed using two different recombinant vectors pPICZαA or pGAPZαB. The expression was controlled by AOX1 or GAP as a promoter, α‐mating factor of *S. cerevisiae* was used to direct the recombinant protein to extracellular medium. Recombinant vectors were linearized by BstXI and transformed into *P. pastoris* KM71H strain. Numerous transformants were generated; RABV‐G gene copy number was determined by real‐time quantitative PCR.

Recombinant clones harboring seven copies of the RABV‐G gene named gap7 and aox7 were used in this work. Empty vectors (pPICZαA and pGAPZαB) were transformed into *P. pastoris* KM71H; selected clones were named as NC (negative control).

YPD agar (2% peptone, 1% yeast extract, 2% glucose, and 20 g L^−1^agar) was used for clone maintenance. BMGY (1 mmol/L potassium phosphate pH 6, 2% peptone, 1% yeast extract, 1.34% yeast nitrogen base, 1% glycerol, and 4 × 10^−5%^ biotin) was used for cell growth before the induction of the aox7 clone. BMMY (1 mM potassium phosphate pH 6, 2% peptone, 1% yeast extract, 1.34% yeast nitrogen base, 1% methanol, and 4 × 10^−5%^ biotin), containing methanol as a carbon source was used to induce the expression of RABV‐G by aox7 clone.

BGY (1 mmol/L potassium phosphate pH 6, 2% peptone, 1% yeast extract, 1.34% yeast nitrogen base, glucose 7.2 g L^‐1^, and 4 × 10^−5%^ biotin) was employed for the expression of RABV‐G by gap7 clone.

### 2.2 Expression of recombinant RABV‐G protein in deep well plates

Recombinant *P. pastoris* clones were grown in YPD agar plates at 30°C. For expression studies, a single colony of selected recombinant clones was used to inoculate 2 ml of BMGY in deep well plate (Dominique Dutscher, France), then grown overnight at 30°C and 250 rpm. After 14–16 hr, optical density was measured at 600 nm, and cells were resuspended in 2 ml of fresh BMMY medium or BGY to an initial OD_600_ of 1. Cultures were performed at 250 rpm 30°C up to 72 hr; Carbon mole (C‐mol) amount was kept similar in both cultures. Methanol and glucose levels were equal to 10 g L^−1^ and 7.2 g L^−1^, respectively; they were added to 1% every 24 hr of culture. Aliquots were taken every 24 hr, cells were pelleted; supernatants and cells were stored at −20°C for further analysis.

### 2.3 Analytical methods

Biomass level was determined by optical density at 600 nm. Yeast cell morphology was determined by direct examination of a drop of recombinant *P. pastoris* clone culture using a light microscope (Leica DFC425, Germany), and 40X objective. Cell size (μm) was estimated on 80 cells using the image‐J software.

Glucose level was estimated by an enzymatic assay kit (Eurodiag, France). Methanol concentration was estimated by Gas chromatography (GC) (Shimadzu, Kyoto, Japan).

### 2.4 Enzyme‐linked immunosorbent assay test (ELISA)

ELISA test was performed to quantify of RABV‐G expressed by the different clones. Detailed protocol was described in Ben Azoun, Belhaj, Göngrich, et al., 2016. Briefly, 100 μl per well of either the sample or the standard (inactivated and purified rabies virus) were incubated for 2 hr at 37°C. Thereafter, monoclonal antibody anti‐glycoprotein TW1 (NIBSC, Hertfordshire, UK) was added to the wells and incubated for 1 hr at 37°C. Finally, anti‐human antibody coupled to peroxidase (Sigma Aldrich) were added and incubated 30 min at 37°C. After tetramethylbenzidine addition, the reaction intensity was measured at 450 nm. OD values higher than 0.150 were considered as positive.

### 2.5 RNA extraction

For transcript quantification, frozen cells were resuspended in Trizol reagent (Invitrogen, CA, USA) and disrupted with glass beads in FastPrepTM cell homogenizer (Thermofisher, MA, USA). Total RNA was then extracted using the RNeasy Kit from Qiagen following the manufacturer's instructions. RNA was tested in 1% agarose gel, and was quantified by measuring OD 230/260/280 using a NanoDrop (Thermo Scientific, MA, USA).

### 2.6 Quantitative real‐time PCR to determine gene transcriptional level

The PCR primer design was conducted using Primer 3 software (http://www.genome.wi.mit.edu/ftp/distribution/software/). qPCR primers used in this work are listed in the supporting information Table S1

RT‐qPCR was performed using a thermal cycler (Bio‐Rad, CA, USA). After preincubation at 95°C for 10 min, the thermal cycler was programmed to perform 45 cycles of: 15 s at 95°C; 20 s at 60°C; 15 s at 72°C. The specificity of amplicons was checked by agarose gel electrophoresis and melting curve analysis after 40 cycles. Individual reactions were carried out in 10 μl containing 5 μl Maxima^™^ SYBR^®^ Green qPCR Master Mix (Roche, Mannheim, Germany), 0.25 μl fw‐ and rev‐ primers and 2.5 μg cDNA.

### 2.7 Transcription levels of RABV‐G and intracellular genes

The mRNA level of RABV‐G and the intracellular genes in each clone was normalized using actin gene as the endogenous control (housekeeping gene). In this case, the transcription levels of RABV‐G gene and intracellular genes in the negative control (NC) (clone transformed with the empty vector) were set as control to normalize the data. RT‐qPCR reactions were run in triplicate with biological replicates (independent experiments) to allow for the statistical confidence in differential gene expression. As amplification efficiencies of the housekeeping reference gene and the target gene were similar, the relative quantification is then determined according to the following equation as described by Dheda et al. (2014).

$$\text{mRNA level}=2^{-\Delta \mathrm{Ct}}$$

where ∆Ct = Ct of target – Ct of reference (Actin gene)

## 3 Results

### 3.1 Cell morphology, growth profiles, and RABV‐G production of recombinant clones of *P. pastoris* expressing RABV‐G

Two recombinant *P. pastoris* clones named gap7 and aox7 harboring seven copies of RABV‐G gene under the control of GAP and AOX1 promoters, respectively, were compared in terms of cell morphology, cell growth, and the expression level of RABV‐G, during glucose and methanol cultivation.

**Figure 1:**
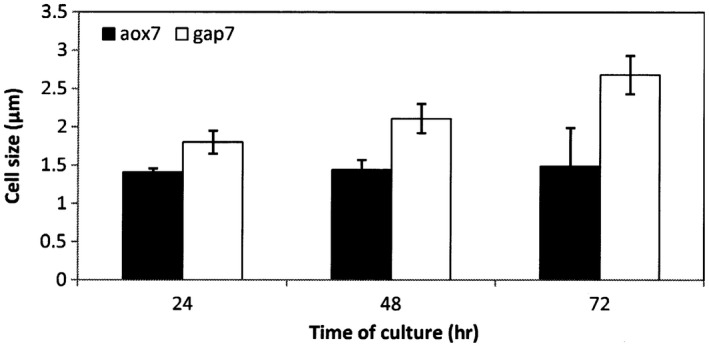
Cell size distribution of aox7 and gap7 recombinant clones throughout culture time. Cells were observed under a light microscope (X40) and cell size was determined by Image‐J software (*p*‐values <.05)

Cell morphology of gap7 and aox7 recombinant clones was analyzed on a complex medium containing different carbon sources, namely BMMY with methanol as sole carbon source, and BGY with glucose as a sole carbon source. Microscopic observations carried out after 24 hr, 48 hr, and 72 hr of culture showed important morphological variations between the two recombinant clones of *P. pastoris* (Figure 1).

After 24 hr of culture, aox7 clone cells grown in BMMY medium containing methanol as the sole carbon source showed a cell size which was 1.27‐fold smaller than the gap7 clone cell size (Figure 1). Cell size difference during both cultures increased over time. At 48 hr of aox7 clone culture, cell size evolved to 1.44 ± 0.13 μm which was 1.46‐fold lower than gap7 clone cell size (2.11 ± 0.19 μm), while at 72 hr of culture, cell size difference became more significant. At this culture time, yeast cells cultivated on methanol showed a cell size around 1.49 ± 0.5 μm, whereas for those grown on glucose, cell size was 2.68 ± 0.25 μm. These results show that aox7 clone cell size was almost constant during the culture, while for gap7 clone, cell size increased by 1.17‐fold and 1.5‐fold at 48 hr and 72 hr, respectively, when compared to 24 hr of culture (Figure 1).

**Figure 2:**
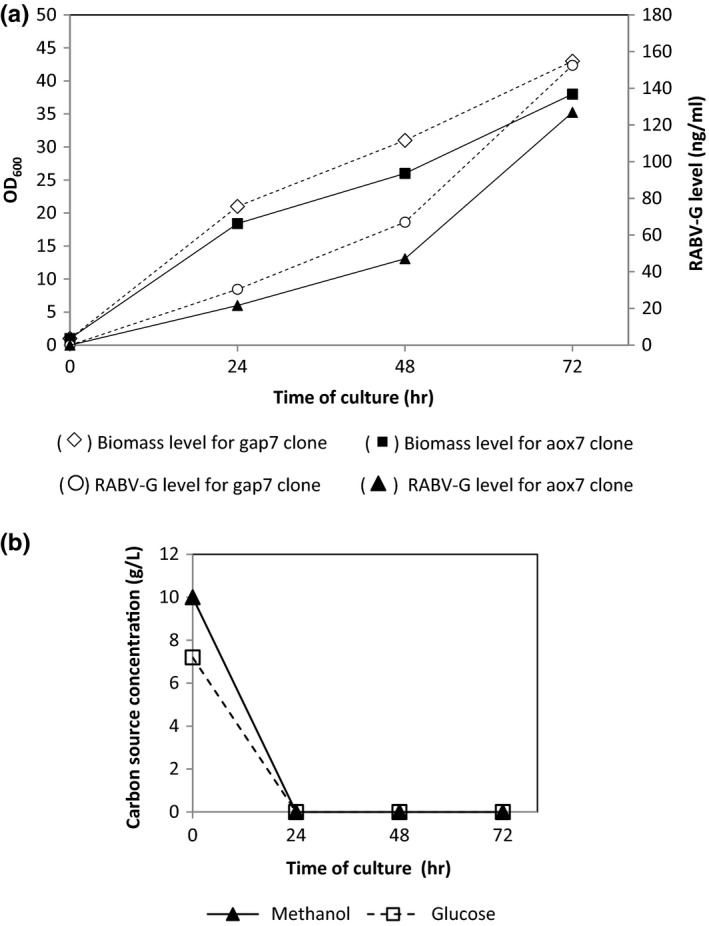
(a) Effect of carbon source on biomass and RABV‐G production level. The gap7 and aox7 clones were cultivated on glucose and methanol, respectively. (b) Residual carbon source concentration during gap7 and aox7 culture on glucose and methanol, respectively

The use of different carbon sources also resulted in different biomass levels; cell growth profile showed a continuous increase for both clones (Figure 2a). For aox7 clone culture, OD_600_ level reached 18 and 38 after 24 hr and 72 hr of induction, respectively. While for gap7 clone, biomass level increased more rapidly, OD_600_ reached 21 at 24 hr and 43 at 72 hr. Final biomass level obtained during gap7 clone culture was slightly higher than that of aox7 clone.

RABV‐G level increased with cell growth in both cultures. For aox7 clone, the highest expression level was obtained at 72 hr and was equal to 127 ng ml^−1^. On the other hand, the secreted protein (RABV‐G) concentration achieved during gap7 clone culture was 1.2‐fold higher (152 ng ml^−1^) than aox7 clone production level.

The difference in RABV‐G production level observed between both clones can not be explained by a difference of the transcriptional level of RABV‐G gene, as no significant difference in RABV‐G mRNA levels was seen (Figure S1). Hence, it appears that this difference is not due to the promoter itself, and that RABV‐G expression depends on culture conditions, especially the carbon source used by the cells.

The difference in biomass level and RABV‐G concentration observed during aox7 and gap7 cultures in different media containing the same carbon molar amount, suggest that cells exhibit a differential use of carbon source. Residual methanol and glucose were not detected in the culture medium for both clones (Figure 2b), showing a full uptake of the carbon source.

Correlating heterologous protein secretion to cell growth is not straightforward and the relation between those parameters is complex. Nevertheless, Buchetics et al. (2011) reported that recombinant clones of *P. pastoris* producing proteins in large amounts are mainly in M and G2 phases of the cell cycle. The authors also reported that overexpression of the main mitotic cyclin *CLB2*, which promotes entry into mitosis and progression from the metaphase to anaphase transition, has improved antibody Fab fragment and human trypsinogen production by 1.32‐ and 2.1‐fold, respectively, in *P. pastoris* recombinant clones.

**Figure 3:**
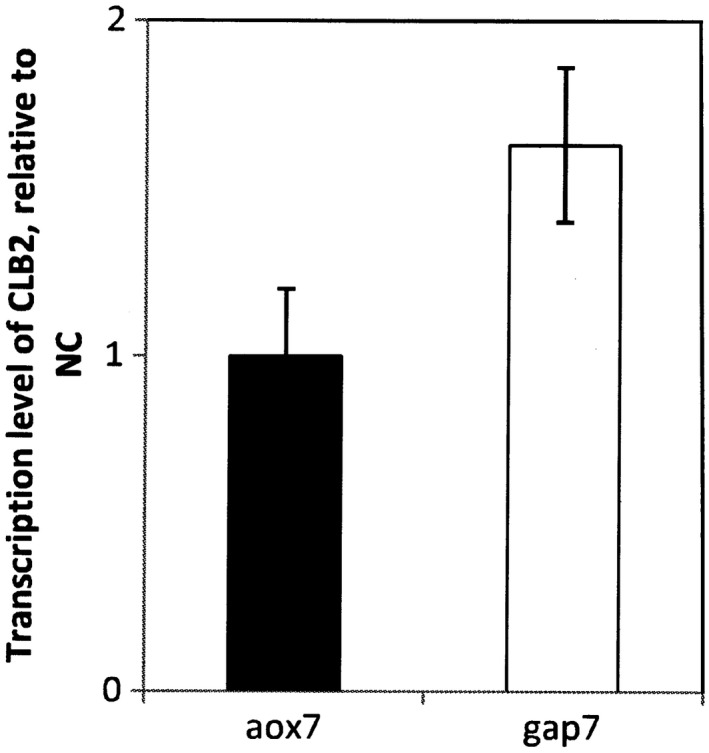
CLB2 transcription level in the aox7 and the gap7 clones relative to the negative control (NC). NC corresponds to *P. pastoris* KM71H strain transformed by pPICZαA or pGAPZαB, and cultivated in the same culture condition as aox7 and gap7 clones

Based on the impact of this protein on cell cycle phase, an analysis of the transcriptional level of the *CLB2* gene was performed by RT‐qPCR in the aox7 and the gap7 clones. Figure 3 showed that the transcript level of *CLB2* gene in the gap7 clone was 1.63–fold higher than the level seen in clone aox7 at 72 hr of culture. This result confirms that a high secretion level of heterologous proteins in *P. pastoris* correlates with a high transcription level of *CLB2* gene and the predominance of cells in G2 and M phases of the cell cycle.

### 3.2 Transcription of central metabolism genes

**Figure 4:**
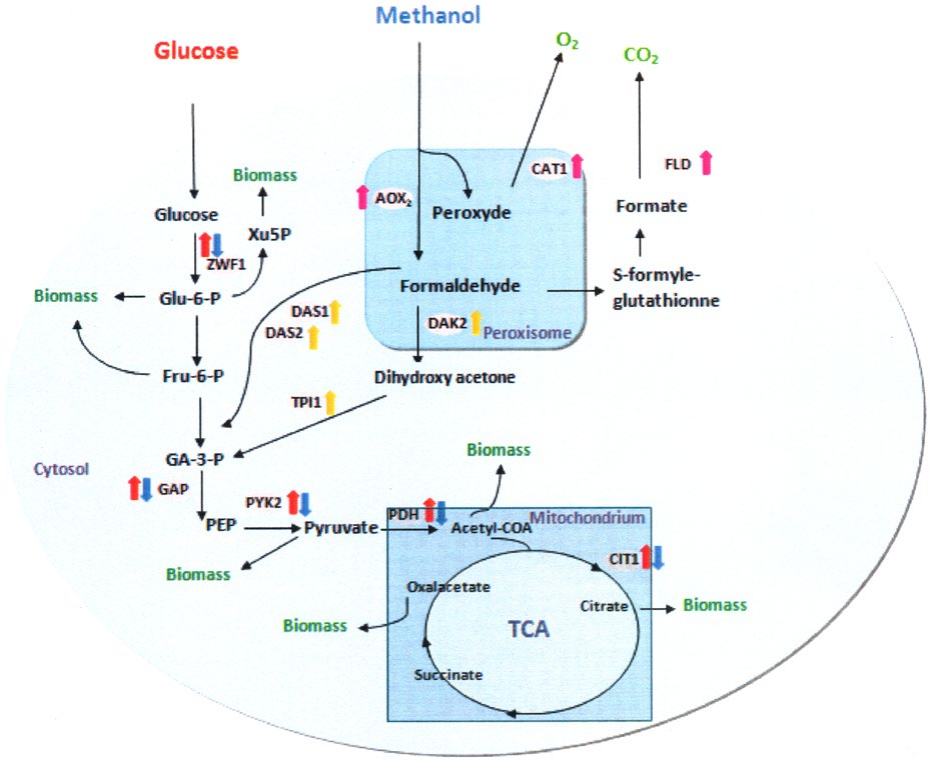
Main changes of transcriptional levels of genes involved in carbon metabolism in the aox7 and the gap7 recombinant clones relative to the negative control (NC). Aox7 and gap7 clones were cultivated on methanol and glucose, respectively. Upward arrows indicate higher mRNA levels and downward arrows indicate reduced mRNA levels. Black arrows indicate genes expressed on glucose and white with continuous and dotted lines arrows indicate genes expressed on methanol. *GAP*: glyceraldehyde‐ 3‐phosphate dehydrogenase, *PYK*: pyruvate kinase, *PDH*: pyruvate dehydrogenase, *CIT1*: citrate synthase, *ZWF1*: glucose‐6‐phosphate dehydrogenase, *AOX2*: alcohol oxidize II,*FLD*: formaldehyde dehydrogenase, *CAT1*: catalase, *DAK*: dihydroxyacetone kinase, *DAS1/2*: dihydroxyacetone synthase, and *TPI1*: triose phosphate isomerase

To obtain better insights into host physiology, especially to identify changes in the central carbon metabolism in different culture conditions, a transcriptional study of key enzymes of the central metabolic pathway for clones cultivated in glucose or methanol‐containing media were investigated (Figure 4). These enzymes include (1) glyceraldehyde‐ 3‐phosphate dehydrogenase (*GAP)* and pyruvate kinase (*PYK)* which are two key enzymes in glycolysis, (2) pyruvate dehydrogenase (*PDH)* which is an acetyl‐CoA‐producing enzyme, (3) citrate synthase (*CIT1)* which is a key enzyme in the TCA cycle, and (4) glucose‐6‐phosphate dehydrogenase (*ZWF1*) the first enzyme of the pentose phosphate pathway.

**Figure 5:**
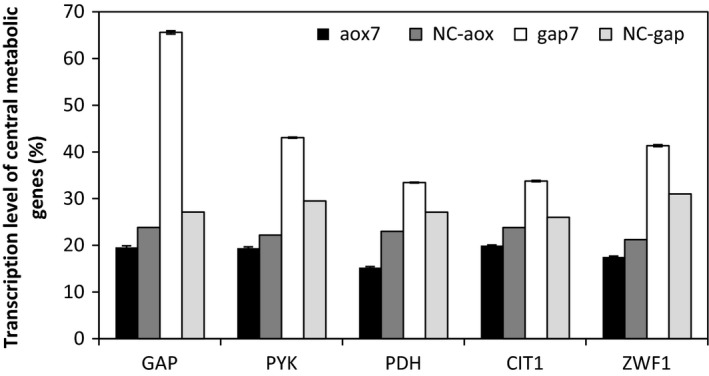
Transcription levels of central metabolic genes in the different clones after 72 hr of cultivation. The aox7 and gap7 recombinant clones were grown on methanol and glucose, respectively. The negative controls: NC‐aox and NC‐gap, correspond to *P. pastoris* KM71H strain transformed by the empty vector pPICZαA or pGAPαB, respectively, and cultivated in the same conditions as aox7 and gap7 clones

A significant difference in the transcription levels of the five genes was observed in the gap7 clone compared to the aox7 clone (Figure 5).

The transcription level of the *GAP* gene was upregulated in the gap7 clone (2.4‐fold) and downregulated in the aox7 clone. In addition, the gap7 clone showed a significant increase in *PYK*,* PDH, CIT1*, and *ZWF1* mRNA levels; the transcript amounts were enhanced by 1.45‐, 1.23‐, 1.29‐, 1.33‐fold, respectively. However, the transcript levels of these genes in the aox7 clone were downregulated by 0.87‐, 0.66‐, 0.83‐, 0.82‐fold, respectively, proving that *GAP* overexpression in the gap7 clone had an effect on the expression of central metabolism genes (Figure 5). These results showed that there is a correlation between the upregulation of the central metabolic gene, high biomass concentration, and high product titer of RABV‐G in the gap7 clone during growth on glucose compared to methanol. It seems that methanol does not offer sufficient carbon and energy to effectively support both cell growth and RABV‐G production. To confirm this hypothesis, the transcription levels of the main genes associated to methanol metabolism were determined.

### 3.3 Transcription of methanol metabolism genes

**Figure 6:**
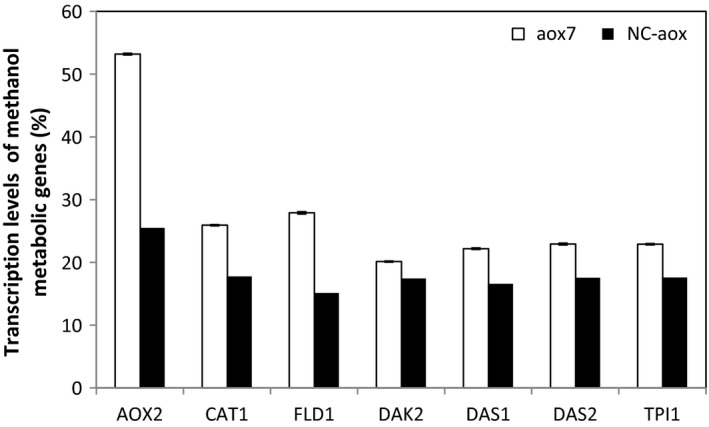
Transcription levels of methanol metabolic genes in the aox7 recombinant and the negative control (NC) after 72 hr of cultivation on methanol. NC corresponds to *P. pastoris* KM71H strain transformed by the empty vector pPICZαA

In order to check if there is a difference between the activation of methanol dissimilatory pathway and assimilatory pathway upon methanol induction, we determined the transcript levels of key genes involved in methanol metabolic pathway after 72 hr of induction of the aox7 clone. Genes used in this study are shown in Figure 4; transcripts of alcohol oxidize II (the second enzyme involved in methanol utilization, *AOX2*), formaldehyde dehydrogenase and catalase (two enzymes involved in methanol dissimilatory pathway, *FLD* and *CAT1* to generate CO_2_ and O_2_, respectively), dihydroxyacetone kinase (*DAK*), dihydroxyacetone synthase (*DAS1/2*), and triose phosphate isomerase (*TPI1*) were determined. These three key enzymes are involved in the methanol assimilatory pathway to provide the constituents for cell synthesis. The transcriptional level of *AOX2* in the aox7 clone was compared with the negative control, a strong increase in *AOX2* transcript level (2.08‐fold) was observed. The transcription level of methanol dissimilatory genes showed similar trends; an increase of 1.84‐fold and 1.5‐fold was seen for *FLD1* and *CAT1*, respectively (Figure 6). However, the transcriptional levels of methanol assimilatory genes (*DAK2*,* DAS1*,* DAS2*, and *TPI1*) were slightly upregulated (1.15‐, 1.33‐, 1.30‐, 1.30‐fold, respectively) in the aox7 clone when compared to the negative control. Hence, the increase in the transcriptional levels of methanol assimilatory genes was lower compared to those of the genes involved in methanol dissimilatory pathway (Figure 6). These results suggest that the dissimilation pathway which competes with the assimilation pathway for carbon flow was enhanced. Thus, methanol being incorporated into biomass might be reduced further; this explains why biomass level of aox7 clone culture was lower than that seen with gap7 clone.

### 3.4 Transcription of antioxidative genes

*P. pastoris* cells can support ER stress to some extent without altering their metabolism. Four main genes associated in oxidative stress were chosen for testing: (1) the transcription factor *YAP1* implicated in oxidative stress response and redox homeostasis; it actives the transcription of genes which encode the antioxidant enzymes.; (2) gamma‐glutamylcysteine synthetase (*GSH1*) is the ubiquitous thiol‐containing reductant that maintains the intracellular redox homeostasis by decreasing cellular disulfide bonds and detoxifying harmful molecules; (3) Glutathione peroxidase (*GPX1*) is a essential enzyme of the antioxidant system, involved in reducing H_2_O_2_ and organic hydroperoxides to H_2_O using reduced glutathione as an electron donor (Margis, Dunand, Teixeira, & Margis‐Pinheiro, 2008); (4) Glutathione reductase (*GLR1*) is intended for the recycling of the redox buffer, it is implicated in the defense of yeast cells against oxidative stress and is regulated by *YAP1*.

**Figure 7:**
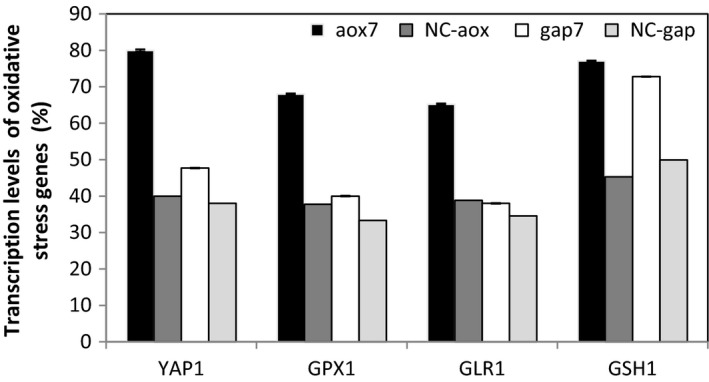
Transcription levels of oxidative stress genes in the different clones after 72 hr of cultivation. The aox7 and the gap7 recombinant clones were grown on methanol and glucose, respectively. Negative controls: NC‐aox and NC‐gap correspond to *P. pastoris* KM71H strain transformed by the empty vector pPICZαA or pGAPZαB, respectively. NC‐aox and NC‐gap were grown on methanol and glucose, respectively

Figure 7 indicates that these antioxidative‐related genes were upregulated at different levels for aox7 clone; the transcript levels of *YAP1, GLR1, GPX1, GSH1* increased by 2‐, 1.68‐, 1.8‐, 1.7‐fold, respectively. However, for gap7 clone grown on glucose, the increase of transcription levels of these genes was lower than that seen for aox7 clone; they increased by 1.26‐, 1.1‐, 1.2‐, 1.45‐fold for the genes *YAP1, GLR1, GPX1, GSH1,* respectively.

A significant increase of *GSH1* gene transcript was observed in both clones (Figure 7). This is due to the oxidative folding of RABV‐G, since both clones contain a high RABV‐G gene copy number.

## 4 Discussion

Although *P. pastoris* is an expression system widely used for protein production, its physiology during heterologous protein expression using different carbon sources has not been extensively studied as it was done for *S. cerevisiae* and *E. coli*. Only few studies describing the effect of central metabolism on recombinant protein production were reported (Çelik et al., 2009; Heyland, Fu, Blank, & Schmid, 2011; Nocon et al., 2014; Prielhofer et al., 2015).

In this work, we studied the effect of carbon source on the physiology of recombinant clones of *P. pastoris* expressing RABV‐G. Cell size is considered as one of the most important determinant of cellular physiology; hence cell size of gap7 and aox7 clones grown on glucose or methanol, respectively, was determined. We showed that cells of gap7 clone cultivated on glucose were larger than those seen on methanol (Figure 1). This result indicates that cell morphology of *P. pastoris* recombinant clone can be affected by the carbon source. Turner, Ewald, and Skotheim (2012) reported that cell size depends on extracellular conditions. In general, growth rate and nutrient conditions (type of carbon source and its concentration) are two key elements that significantly affect cell size. Yeast cells cultivated on a rich medium manifest a larger size, whereas those growing on a poor medium show a slow growth and a smaller cell size. Cipollina et al. (2008) reported that cell switch from one medium condition to another containing, for example, a different carbon source, influences cell size. When cells of *S. cerevisiae* were removed from ethanol (slow growth) to glucose (fast growth) they rapidly adapt to a larger size. Pluskal, Hayashi, Saitoh, Fujisawa, and Yanagida (2011) also showed that when a deficiency in glucose occurs, *S. pombe* undergoes several cycles of division without growing, to achieve a low size. Thus, there is a close and complex relationship among cell morphology and cell growth in yeasts.

In this study, glucose and methanol as a carbon source were compared during the culture of two recombinant clones expressing RABV‐G. Glucose seems to be a better carbon source than methanol, resulting in higher biomass concentration, and higher RABV‐G yield (Figure 2). However, comparable specific growth rates (average specific growth rate for both clones around 0.04 hr^−1^) were obtained for both clones which is contrary to data described in the literature. Gap7 clone should exhibit a higher specific growth rate than aox7 clone. Nevertheless, gap7 clone showed a specific productivity of RABV‐G which was 20% higher than that obtained with aox7 clone, probably because of the higher transcriptional levels of the genes involved in the central metabolic pathway as explained below. Generally, it is well recognized that cell growth rate has an important role in gene regulation, and therefore in protein production (Liang et al., 2014). It appears that a high growth rate favors protein production in *P. pastoris* since genes involved in gene translation and expression are overexpressed. On the other hand, Buchetics et al. (2011) reported that *P. pastoris* recombinants clones exhibiting a high secretion level are mostly in the G2 and M phases of cell cycle. The relation between cell cycle phase and protein production has been described not only in yeasts (Frykman & Srienc, 2001) but also in mammalian cells (Charlet, Kromenaker, & Srienc, 1995). The eukaryotic cell cycle has been examined comprehensively in the model organism *S. cerevisiae* reviewed in Cid et al. (2002).

The appropriate progress of cell cycle is monitored by interferences of cyclins and cyclin‐dependent kinases (*CDK*) and related regulators and phosphatases. Buchetics et al. (2011) showed that upregulation of cyclin gene *CLB2* of *P. pastoris* led to the expected cell cycle shift toward a higher fraction of cells in the G2/M phase, and subsequently increased the product yield. In this study, the *CLB2* transcript level in the two clones during their cultivation on glucose or methanol was determined. Interestingly RT‐qPCR data showed that mRNA level of *CLB2* in the gap7 clone cultivated on glucose were 1.63‐fold higher than in the aox7 clone cultivated on methanol after 72 hr of culture. A higher fraction of gap7clone cells in the G2/M phase compared to aox7 clone would probably be observed.

Buchetics et al. (2011) also showed that cell cycle phase impacts protein secretion; co‐overexpression of *CLB2* mitotic cyclin in recombinant *P. pastoris* clones resulted in the enhancement of the production levels of human trypsinogen and Fab proteins. Thus, it seems that high level secretion of heterologous proteins correlates with the overexpression of the *CLB2* mitotic cyclin. It would be sound to verify if this hypothesis can be applied as a general rule to enhance recombinant protein expression in *P. pastoris*.

Our results also show that co‐overexpression of *CLB2* is expected to increase the expression level of RABV‐G in *P. pastoris*. Overexpression of other mitotic cyclins like *CLB4* can also improve RABV‐G expression in this yeast, as shown for other proteins (Buchetics et al., 2011).

In this work, the main enzymes of the central carbon metabolism pathway were studied. The aim was to investigate the effect of the carbon source on the physiology of recombinant clones of *P. pastoris*.

Glycolysis pathway starts with glucose conversion into glucose‐6‐phosphate which can then be converted into biomass or to fructose‐6‐phosphate. In the case of methanol, the central carbon metabolism is activated from glyceraldehyde‐3‐phosphate dehydrogenase (*GAP*) to generate the energy necessary for cell growth and protein production (Çelik et al., 2009). The expression of genes related to glycolysis such as *GAP* and *PYK* in the gap7 and the aox7 clones are shown in Figures 4 and 5. We found that the transcription levels of glycolysis genes in cells growing on methanol were lower compared to those cultivated on glucose. Interestingly, similar result was observed in a recombinant clone of *P. pastoris* grown on methanol and expressing porcine insulin precursor (Zhu, Guo, Zhuang, Chu, & Zhang, 2011).

The transcription levels of glucose‐6‐phosphate dehydrogenase (*ZWF1*), a key enzyme in the pentose phosphate pathway, pyruvate dehydrogenase (*PDH)* which is an acetyl‐CoA producing enzyme, and citrate synthase (*CIT1)* which is one of the enzymes of the TCA cycle were determined. All these genes were upregulated on glucose but downregulated on methanol. Therefore, it appears that there is a correlation between the upregulation of the central metabolic genes, high biomass concentration, and high product titer during *P. pastoris* growth on glucose compared to methanol. Prielhofer et al. (2015) showed that various *P. pastoris* genes encoding enzymes implicated in the metabolism of carbon sources are transcribed differently depending on the carbon source used, they found that *P. pastoris* glycolytic genes were downregulated during culture on limiting level of glucose, glycerol, and methanol compared to an excess of glucose. However, the genes encoding the gluconeogenic enzymes were downregulated on excess glucose compared to the other conditions. Nocon et al. (2014) reported that the central metabolism pathway especially the pentose phosphate pathway (PPP) affects the production level of cytosolic human superoxide dismutase in *P. pastoris*. They also showed that coexpression of ZWF1gene had a positive effect on intracellular production of human superoxide dismutase and bacterial ß‐glucuronidase in *P. pastoris* (Nocon et al., 2014). Our study suggests that overexpression of *ZWF1* would also improve the expression level of heterologous proteins targeted to secretion in this yeast. It will be also useful to study if co‐overexpression of the 6‐gluconolactonase (*SOL3*), the second enzyme in the PPP would improve the secretion of RABV‐G, as demonstrated for intracellular expression by Nocon et al. (2016).

Methanol metabolism is another remarkable physiological change in the aox7 clone. After 72 hr of induction, the transcript levels of genes involved in methanol dissimilatory pathway (*FLD1* and *CTA1*) were highly induced compared to genes involved in assimilatory pathway (*DAS1/2*,* DAK2*,* TPI1*). These observations correlate with those described by Zhu et al. (2011), they showed that the genes involved in dissimilatory pathway were upregulated in high copy clones expressing porcine insulin precursor (PIP) under the control of AOX1 promoter. The significant activation of methanol assimilation pathway compared to central carbon metabolism pathway could be the reason for reduced RABV‐G protein production in the aox7 clone compared to gap7 clone, particularly when we take into account that RABV‐G transcript level was similar for both clones (Ben Azoun, Belhaj, Göngrich, et al., 2016; Ben Azoun, Belhaj, & Kallel, 2016). These data indicate that methanol does not offer enough carbon and energy to strongly sustain cell growth and RABV‐G production. One way to alleviate these limitations could be the co‐overexpression of the proteins involved in methanol assimilation pathway.

Methanol catabolism generates the production of H_2_O_2_, needing the action of antioxidants. *YAP1*, the oxidative stress response transcription activates the expression of genes related to the glutathione redox system such as *GLR1, GPX1*, and *GSH1*. This study showed that these genes were significantly highly expressed in cells growing on methanol compared to those growing on glucose. The upregulation of *GSH1* transcript level was observed in both conditions suggesting that cysteine was limited in both recombinant clones (Nisamedtinov et al., 2011).

The rise of oxidative stress genes in the aox7 clone indicates that cellular redox potential gradually shifted to a more oxidizing state throughout culture on methanol. An explanation might be that the antioxidant gene GSH1 was upregulated during RABV‐G production on methanol, due to oxidative folding of the foreign protein, meaning that the cells become highly exposed to formaldehyde accumulation. Thus, *P. pastoris* induced the up‐expression of *FLD1* to detoxify formaldehyde (Sunga & Cregg, 2004; Zhu et al., 2009) and to maintain healthy redox balance in this metabolic state.

In conclusion, in this study, we studied the impact of glucose or methanol as a carbon source on the physiology of *P. pastoris* recombinant clones expressing RABV‐G through the transcriptional analysis of genes involved in carbon metabolism pathways. Our results suggest that when glucose was used, an increase in biomass and RABV‐G production levels were obtained when compared to the recombinant clone grown on methanol. Furthermore, the use of methanol as the sole carbon source resulted in a raise of oxidative stress. Hence, glucose appears to be the preferred carbon source for the expression of RABV‐G in *P. pastoris* and can be utilized to set‐up the high cell density production process in a bioreactor.

## Conflict of Interest

The authors declare that they have no conflict of interest.
