# Supplementary material for: Investigating the effect of carbon source on rabies virus glycoprotein production in Pichia pastoris by a transcriptomic approach
Source: Microbiologyopen. 2017 May 18;6(4):e00489. doi: 10.1002/mbo3.489 (PMC5552951; doi:10.1002/mbo3.489)
Supplement: Supplementary file 1 [file MBO3-6-na-s001.docx]

Fig. S1

Fig. S1 Transcription levels of RABV-G gene in the aox7 and the gap7 recombinant clones relative to the clones with one copy of RABV-G gene, aox1 and gap1, respectively.
