# Supplementary material for: Investigating the effect of carbon source on rabies virus glycoprotein production in Pichia pastoris by a transcriptomic approach
Source: Microbiologyopen. 2017 May 18;6(4):e00489. doi: 10.1002/mbo3.489 (PMC5552951; doi:10.1002/mbo3.489)
Supplement: Supplementary file 2 [file MBO3-6-na-s002.docx]

Table S1 : Oligonucleotide sequences used in this study

| Primers | Sequence (5’-3’) |
| --- | --- |
| Actin-F | CCTGAGGCTTTGTTCCACCCATCT |
| Actin-R | GGAACATAGTAGTACCACCGGACATAACGA |
| RABV-G-F | TCCATTGGCTGATCCATCTACTG |
| RABV-G –R | CCAGTTTGGCAAACCCAGATC |
| GPX1-F | CTATTGTCGGATTTCCCTGTAACCAGTTT |
| GPX1-R | TCCGAACCGTTGACATCAATCTTTTT |
| GLR1-F | CAAGGGGACAATGAGAAAGTGGTT |
| GLR1-R | CAGGTCCTCGTTGGTGAAGA |
| YAP1-F | CAGGCCAACTACCGTCACCAACTTCTA |
| YAP1- R | AGCCATCCACAGACTCATCAAAT |
| GSH1-F | AGAGGCAACACCAGCTTCTC |
| GSH1- R | GGGAAGACAGTCAGCGTCAA |
| GAP-F | ATGACCGCCACTCAAAAGAC |
| GAP- R | GCACCAGTGGAAGATGGAAT |
| PYK- F | CAAGTGCAATCTGGCAGGTA |
| PYK–R | GCATCATAGCAACAGCCTCA |
| PDH-F | ATGGGTTTCACTGGTCTTGC |
| PDH-R | ACAGGGTTGCTTACCACCAG |
| CIT1-F | TTCAAAGACGGAAAGGTTGG |
| CIT1-R | CAACCAAGTGAGTGGTGTGG |
| ZWF1-F | CGACTTGCAGTCAGCAGAAG |
| ZWF1-R | TGATGAATGCGTTTCCAAAA |
| AOX2-F | GTGCTAACGTCAGGGTTGTTG |
| AOX2-R | CGTGAATGTCAGGGTTGTTG |
| CTA1-F | AGGTTTGGCCACACAAAGAC |
| CTA1-R | TGAAGGACTGGATCAGCAGATG |
| FLD-F | TATCAACACCACCGCCAATG |
| FLD-R | TGGTACAAAGCGGGGTTATAGG |
| DAK2-F | ACACACTGACCCTTGAAACG |
| DAK2-R | TCATGGGCAGCTTTGTTTGC |
| DAS1-F | AACGGTCTATCTGCCGTAGATG |
| DAS1-R | GGAAAAGAGCCTCAACCAATGG |
| DAS2-F | ACACCCCCTCAATCTTCAGATG |
| DAS2-R | AGCAGTGTTTCCTGCAAAGC |
| TPI1-F | TGCTCTTGACCAGGGTTTGA |
| TPI1-R | AGTGGCAGCAAGACCAGTAC |
| CLB2-F | AAAGTGCTCCCGGAAGAACA |
| CLB2-R | GTTGGTGGCCTCTCAGAACT |

F : Forword R : Reverse
